# Inflammation and Corticospinal Functioning in Multiple Sclerosis: A TMS Perspective

**DOI:** 10.3389/fneur.2020.00566

**Published:** 2020-07-07

**Authors:** Mario Stampanoni Bassi, Fabio Buttari, Luana Gilio, Nicla De Paolis, Diego Fresegna, Diego Centonze, Ennio Iezzi

**Affiliations:** ^1^Unit of Neurology & Neurorehabilitation, IRCCS Neuromed, Pozzilli, Italy; ^2^Laboratory of Synaptic Immunopathology, IRCCS San Raffaele Pisana, Rome, Italy; ^3^Laboratory of Synaptic Immunopathology, Department of Systems Medicine, Tor Vergata University, Rome, Italy

**Keywords:** Transcranial magnetic stimulation (TMS), multiple sclerosis (MS), inflammation, synaptic transmission, cytokines

## Abstract

Transcranial magnetic stimulation (TMS) has been employed in multiple sclerosis (MS) to assess the integrity of the corticospinal tract and the corpus callosum and to explore some physiological properties of the motor cortex. Specific alterations of TMS measures have been strongly associated to different pathophysiological mechanisms, particularly to demyelination and neuronal loss. Moreover, TMS has contributed to investigate the neurophysiological basis of MS symptoms, particularly those not completely explained by conventional structural damage, such as fatigue. However, variability existing between studies suggests that alternative mechanisms should be involved. Knowledge of MS pathophysiology has been enriched by experimental studies in animal models (i.e., experimental autoimmune encephalomyelitis) demonstrating that inflammation alters synaptic transmission, promoting hyperexcitability and neuronal damage. Accordingly, TMS studies have demonstrated an imbalance between cortical excitation and inhibition in MS. In particular, cerebrospinal fluid concentrations of different proinflammatory and anti-inflammatory molecules have been associated to corticospinal hyperexcitability, highlighting that inflammatory synaptopathy may represent a key pathophysiological mechanism in MS. In this perspective article, we discuss whether corticospinal excitability alterations assessed with TMS in MS patients could be useful to explain the pathophysiological correlates and their relationships with specific MS clinical characteristics and symptoms. Furthermore, we discuss evidence indicating that, in MS patients, inflammatory synaptopathy could be present since the early phases, could specifically characterize relapses, and could progressively increase during the disease course.

## Introduction

Multiple sclerosis (MS) is an inflammatory immune-mediated disease of the central nervous system (CNS) with white matter demyelinating lesions and chronic diffuse neuronal degeneration, causing variable and unpredictable clinical manifestations and disease course.

Transcranial magnetic stimulation (TMS) is a neurophysiological technique that exploits the principles of electromagnetic induction. A coil of wire, connected to an electric pulse generator and placed over the scalp, produces a strong magnetic pulse of very short duration able to penetrate through the intact skull, inducing an electric current in the underlying neural tissue noninvasively and almost painlessly (1). The induced electric current mainly flows tangentially to the brain surface, preferentially activating cortical fibers oriented in parallel to the electric field (2). When applied over the primary motor cortex (M1), TMS excites the corticospinal system, eliciting multiple descending volleys, which reflect both the direct activation of cortical motor axons (D-waves) and the indirect, trans-synaptic activation of motor cortical neurons (I-waves) (3, 4). The recruitment of different combinations of D- and I-waves depends on the stimulus intensity, pulse configuration, coil shape, and orientation (5–9). In particular, with a posterior-to-anterior induced current flow, TMS at lower intensities evokes I-waves, whereas at higher intensities also D-waves occur (10). These descending activities can be recorded in contralateral target muscles as motor-evoked potentials (MEPs).

TMS is used in the clinical context of MS together with multimodal evoked potentials (i.e., visual and somatosensory) as a useful tool to detect subclinical involvement of the corresponding functional system with the aim to help early diagnosis (11). TMS alterations have also been correlated to demyelinating damage and neuronal degeneration in different MS phenotypes. For example, slowed central motor conduction time (CMTC) and reduced MEP amplitude can indicate axonal depletion or even extreme asynchrony of the multiple descending volleys to spinal motoneurons due to conduction blocks in the myelinated fibers along the corticospinal tracts (12, 13).

Experimental evidence from studies in animal models and in patients with MS suggests that inflammation critically affects synaptic functioning (14). Accordingly, neurophysiological alterations have been detected even in the absence of macroscopic damage, suggesting a role of additional pathological mechanisms (15). In particular, different proinflammatory and anti-inflammatory molecules can influence cortical excitability in MS (16) representing an additional cause of impaired synaptic functioning.

In this perspective article, we provide an overview of the main TMS studies exploring corticospinal excitability and connectivity alterations in MS, their pathophysiological correlates, and their relationship to clinical characteristics and symptoms. In addition, evidence from preclinical data and TMS studies, which highlight the role of inflammatory synaptopathy as a relevant pathophysiological mechanism that acts since the early phases of MS, is discussed.

### TMS as a Tool to Investigate Cortical Excitability in MS

TMS can be used to assess the functionality of the corticospinal tract and the corpus callosum (CC) and to explore some physiological properties of M1. Various TMS paradigms have been designed to investigate corticospinal excitability to test excitatory and inhibitory interactions in M1 and to probe M1 connectivity (Table 1). Single-pulse TMS can be used to assess simple cortical excitability measures, such as motor thresholds, to study MEP characteristics, to estimate CMCT, and to test cortical inhibition. With paired-pulse TMS, it is possible to explore specific inhibitory and excitatory circuits in M1. During paired-pulse TMS, two stimulators are connected to the same coil that delivers two consecutive pulses at variable interstimulus intervals (ISIs). In addition, TMS has been used to investigate interhemispheric effective connectivity of M1 by exploring transcallosal connections with either single or double-coil (d-c) approaches. In d-c TMS, two stimulation units, each connected to a corresponding coil, are used to target different motor cortical regions at various ISIs.

**Table 1:** Main TMS protocols used to explore motor cortex pathophysiology in MS patients.

SINGLE-PULSE TMS	**Motor thresholds**	Resting and active motor thresholds (RMT and AMT), tested with single-pulse TMS in resting and contracted muscles respectively, represent simple measures of the excitability of the whole corticospinal system, including the fluctuating excitability of both M1 pyramidal cells and spinal motoneurons (9) MTs are defined as the minimum intensity of M1 stimulation able to elicit MEPs in the target muscles (9) and MTs likely depend on the axonal excitability regulated by voltage-gated sodium channels and on the activity of AMPA receptors (17).
	**MEP amplitude**	Commonly used for testing the excitability of the whole corticospinal system. TMS activates along the corticospinal tracts a series of descending volleys with different thresholds, different conduction velocities, and intrinsic asynchrony of propagation (8, 10). Temporal dispersion is further enhanced by peripheral nerve conduction, leading to phase cancellation of motor unit action potentials (18, 19). MEP amplitude can be influenced by excitability changes occurring at cortical level, representing an important marker of synaptic activity in the motor cortex (20). Spinal motoneurons excitability also contributes to MEP amplitude. (8, 21).
	**Central motor conduction time (CMCT)**	Represents the time interval elapsing between the cortical stimulus and the arrival of the excitatory input to the spinal motoneurons, being a useful tool to assess the integrity of fast-conducting motor pathways in the corticospinal tract (9). CMCT evaluated with TMS includes the trans-synaptic activation of cortical motoneurons through the chain of cortical interneurons responsible for the I-waves generation. CMCT is commonly calculated by subtracting the peripheral motor conduction time from the MEPs latency (9).
	**Cortical silent period (CSP)**	Inhibitory phenomenon measured in contracting muscles as an interruption of the ongoing voluntary electromyographic activity. Spinal inhibitory mechanisms contribute to the first part of CSP, whereas the late part originates at cortical level (22–24) and expresses GABA-B mediated inhibition in M1 (25–27). The role of GABA-A receptors has also been suggested. In particular, GABA-A receptors could be activated by low stimulus intensity, whereas GABA-B receptors are engaged with stronger pulses (28).
PAIRED-PULSE TMS	**Short-interval intracortical inhibition (SICI)**	SICI is tested with a subthreshold conditioning stimulus followed by a suprathreshold test stimulus at an ISI of 2-5 ms (29). The conditioning stimulus suppresses the excitatory response to the subsequent suprathreshold stimulus (29, 30) depending on GABA-A receptor activity (30–32).
	**Intracortical facilitation (ICF)**	ICF is evaluated with a similar protocol used for SICI with longer interstimulus intervals at 7–20 ms (29, 33). ICF engages M1 circuits different from those involved in SICI with a resulting excitatory effect that combines a weak GABA-A-mediated inhibition and a predominant NMDA-mediated facilitation (33, 34).
	**Short-interval intracortical facilitation (SICF)**	SICF is measured with a particular paired-pulse TMS protocol that uses at very short ISIs either a conditioning suprathreshold stimulus followed by a test subthreshold stimulus (34, 35) or two near threshold pulses (36, 37) and reflects facilitatory I-wave interaction within M1 (35). Pharmacological studies have suggested that SICF is modulated by a number of neurotransmitter systems, including GABA, dopamine, noradrenaline [for reviews, see (35, 38)].
TRANSCALLOSAL CONNECTIVITY	**Ipsilateral silent period (iSP)**	Tests the inhibitory influences existing between the two M1s and is mediated by fibers passing across the corpus callosum (39–41). Refers to the suppression of ongoing voluntary electromyographic activity in hand muscles in response to a single suprathreshold pulse over the ipsilateral M1 likely mediated by GABAergic transcallosal projections (42–44).
	**Interhemispheric connectivity**	Interhemispheric inhibition (IHI) studied with d-c TMS, refers to the suppression of MEPs following suprathreshold conditioning stimuli given over the contralateral M1 (43, 45). IHI is mediated by excitatory inputs coming from the conditioned M1, traveling across the corpus callosum, and reaching local inhibitory synapses in the contralateral target M1 (43, 46, 47) are mediated by GABA-B (48–50). Facilitatory interhemispheric connections have also been studied between dorsal premotor cortex and contralateral M1 (51–53).

#### TMS as a Tool to Investigate Different Pathophysiological Mechanisms in MS

Considering the clinical impact of corticospinal system lesions, different TMS studies have shown several alterations of M1 excitability and corticospinal tract conduction in MS patients. In particular, reduced MEP amplitude (54–56), increased MEP latency (57), and duration (58) have been reported in MS patients compared with control subjects. In addition, increased RMT (55, 59) and prolonged CMCT (54, 59–62) have been frequently evidenced in patients with MS. Overall, these findings have been interpreted in the light of demyelinating conduction block and axonal damage. In fact, demyelination and conduction blocks could lead to a greater temporal dispersion of the corticospinal volleys, resulting in reduced amplitude and increased MEP duration, prolonged MEP latency, and increased CMCT. Conversely, axonal loss could be more relevant in progressive MS, being associated with higher RMT, reduced MEP amplitude, and longer CMCT (55).

Cortical inhibition tested with single-pulse TMS has documented prolonged CSP duration in RR-MS patients (57, 63). One study has shown that, in remitting patients, CSP prolongation was correlated with white matter lesion volume but not with cortical thickness (57). In progressive MS patients, reduced CSP duration correlated with lower whole-brain cortical magnetization transfer ratio (MTR), suggesting a role of cortical damage (56). Altered GABA transmission could explain the CSP alteration although alternative mechanisms have been suggested, including changes in spinal motoneuron excitability (23, 24), attentional processes (64, 65), and altered voluntary motor drive (66). In addition, reduced CSP duration after a fatiguing motor task has been reported in MS patients compared to controls (67), suggesting that additional mechanisms could also be involved.

Various studies have explored SICI in MS patients (55, 56, 68). Although some authors reported comparable SICI between RR-MS and controls (55, 57, 69), in one study it has been found that lower SICI in patients with RR-MS was correlated with reduced MTR in the hand motor cortex (56). In addition, reduced SICI and increased ICF have been reported in SP-MS patients compared with RR-MS and controls (55, 69). It has been proposed that the clinical course of progressive MS phenotypes could be characterized by a deterioration of SICI over time (70). These alterations may reflect hyperexcitability due to enhanced glutamatergic transmission and reduced inhibition, which could be particularly noticeable in progressive patients, being associated with higher disability and cortical atrophy (55).

As the CC involvement represents a hallmark of MS, TMS has been specifically used to test interhemispheric connectivity in these patients. Increased iSP latency and duration have been reported in MS patients compared to controls (60–62, 71). In particular, iSP alterations found in MS have been associated to CC volume (62). One study in MS patients, combining TMS and fMRI, has demonstrated that increased ipsilateral M1 activation during the execution of a motor task was correlated with reduced iSP duration and with ultrastructural damage of the CC (61). However, prolonged iSP duration has also been associated with CMCT prolongation without significant correlations with CC abnormalities, suggesting that transcallosal inhibition could be affected by demyelination of the contralateral corticospinal tract (72). Notably, reduced IHI has also been observed in early RR-MS patients in the absence of macroscopic damage of the CC or CMCT alterations (73). Finally, one TMS study has shown that excitatory interhemispheric connectivity between premotor cortex and contralateral M1 could be reduced, irrespective of CC lesion load and in the absence of disability (53). Although the pathophysiological mechanisms underlying altered interhemispheric connectivity in MS are not fully understood, it is likely that, alternatively to CC structural damage, other mechanisms could be involved, including reduced excitatory projections from the conditioning cortex or defective GABAergic signaling in target M1 inhibitory interneurons (48, 74).

#### TMS Alterations Could Be Associated With Specific MS Clinical Characteristics

Alterations of various TMS measures have been related to MS clinical characteristics. Expanded disability status scale (EDSS) score has been associated with increased RMT, altered MEPs, and prolonged CMCT and iSP duration (55, 58, 60). A positive correlation between these TMS measures and clinical scores could reflect the prevalent role of white matter lesions in the pathogenesis of these alterations, particularly of the corticospinal tract and the CC. The role of white matter disconnection has been specifically involved in cerebellar symptoms. Cerebellar tremor in MS has been associated with lacking cerebellar-M1 inhibitory connectivity tested with d-c TMS (75). In addition, cerebellar dysfunction in MS has also been associated with increased CSP duration, likely resulting from impaired cerebellar projections to M1 (63).

Altered balance between cortical excitation and inhibition in MS has been correlated with clinical severity. One study showed that prolonged CSP duration was correlated with clinical disability and predicted greater motor impairment, suggesting that increased inhibition could lessen clinical compensation, possibly interfering with plasticity (57). Moreover, one study has demonstrated that defective SICF was correlated with increased EDSS in MS patients (76). Alterations involving both inhibitory and excitatory circuits would suggest a specific role of synaptic dysfunction in addition to demyelination of white matter tracts. The finding that steroid administration in relapsing RR-MS led to motor improvement, along with reduced SICI and enhanced ICF (77), supports this hypothesis, suggesting a restored synaptic functioning within M1. Finally, it has been proposed that corticospinal excitability asymmetry between the two hemispheres could represent a marker of clinical disability, whose mechanisms are not completely elucidated and possibly involving neurodegenerative and inflammatory processes (78).

Fatigue represents a frequent and severely disabling symptom in MS patients (79). Different mechanisms have been postulated, including white matter and cortical lesions, endocrine alterations, and the influence of neuroinflammation on brain functioning (80, 81). Enhanced GABAergic activity tested with SICI and CSP has been specifically implicated in MS fatigue (82, 83). In line with the hypothesis of increased M1 inhibition in fatigued MS patients, one study has demonstrated that a fatiguing motor task was associated with increased CSP duration. Notably, unlike healthy controls, CSP alteration also involved untrained adjacent muscles, suggesting that mechanisms of cortical spreading could intervene in generating fatigue in MS (67).

Cognitive dysfunction represents an important symptom frequently underestimated in MS patients, which involves various domains, including executive functions, processing speed, and working memory. In addition to demyelination and gray matter atrophy, different pathophysiological mechanisms, including the presence of cortical lesions, impaired brain network organization, and altered synaptic functioning, have been proposed (84). Short-latency afferent inhibition (SAI), a TMS protocol exploring the efficiency of cortical cholinergic inhibitory activity mediated by peripheral somatosensory afferent inputs to M1 (85), has been used to investigate cognitive dysfunction in MS. In particular, verbal memory impairment was associated with reduced SAI that could be partly reversed by rivastigmine administration (86). Notably, these results are in line with studies demonstrating altered SAI in patients with Alzheimer's disease (87). Although mood disturbances are frequently observed in MS, correlations with TMS alterations have been scarcely investigated. One study showed that anxiety in MS patients was associated with altered inhibitory interhemispheric connectivity, highlighting the role of increased transcallosal transfer (88).

## Inflammatory Synaptopathy as a Link Between Autoimmunity and Disease Manifestations in MS

In MS, auto-reactive T lymphocyte infiltration into the CNS and activation of resident immune cells lead to demyelinating lesions and axonal damage. Inflammatory cytokines released by immune cells play a crucial role in inducing and maintaining the inflammatory response in MS. Proinflammatory molecules promote T-helper 1 (Th1) and Th17 differentiation and lymphocyte activation and migration across the blood brain barrier (89). Accordingly, enhanced expression of various cytokines, including interleukin (IL)-1β, tumor necrosis factor (TNF), IL-6, IL-17, and interferon (IFN)-γ has been reported in animal models (i.e., experimental autoimmune encephalomyelitis, EAE) and in the perivascular infiltrates and cerebrospinal fluid (CSF) of MS patients (90–94).

In addition to their immunomodulatory activity, cytokines modulate the function of oligodendrocytes, astrocytes, and neurons (95, 96). Experimental studies have shown that inflammatory molecules specifically influence synaptic functioning, suggesting that chemokines and cytokines may represent an important communicating system in the CNS. In turn, astrocytes, endothelial cells, and neurons participate in cytokine production (97, 98), generating a neuro-immune crosstalk with crucial roles in physiological and pathological conditions (99, 100).

### Preclinical Studies and Translational Models

Experimental studies have contributed to demonstrate that inflammation alters synaptic functioning (14, 101). In the striatum of EAE mice, electrophysiological recordings revealed enhanced glutamatergic transmission and excitotoxic neurodegeneration occurring since the early phases, before the onset of symptoms, and independently of demyelinating damage (14). These excitotoxic alterations were mainly caused by increased activity and expression of the a-amino-3-hydroxy-5-methyl-4-isoxazolepropionicacid (AMPA) receptor; accordingly, the administration of inhibitors of glutamate AMPA receptors ameliorated the course of EAE and reduced loss of dendritic spines (14). In the same study, TNF released by activated microglial cells was identified as mainly responsible for these alterations as incubation of this molecule reproduced *in vitro* both altered AMPA activity and neuronal damage. Other inflammatory cytokines have been associated with synaptic hyperexcitability in EAE. The proinflammatory cytokine IL-1β induced pathologically enhanced glutamatergic transmission in the cerebellum of EAE mice, reducing glutamate reuptake by altering the expression of the glutamate-aspartate transporter/excitatory amino acid transporter 1 (GLAST/EAAT1) (102). Notably, the administration of the GLAST/EAAT1 inhibitor reproduced the synaptic modifications observed in symptomatic EAE mice (103). In addition, administration of the IL-1 receptor antagonist, a physiological inhibitor of IL-1β (104), ameliorated the course of EAE by reducing astroglia activation and restoring GLAST/EAAT1 expression (102, 105). Proinflammatory cytokines have also been consistently associated with altered inhibitory transmission in EAE mice. It has been evidenced that incubating IL-1β and TNF in mice brain slices impaired GABAergic transmission and promoted excitotoxic neuronal damage (106, 107). Accordingly, enhancing GABA signaling significantly improved the clinical symptoms of EAE, likely as a result of a direct neuroprotective effect and inhibition of inflammatory response (108).

Translational experiments confirmed that a similar subset of proinflammatory molecules mediates synaptic alterations in human MS. One study has demonstrated that the CSF collected from patients with active MRI lesions pathologically enhanced excitatory postsynaptic currents when incubated on mice brain slices, inducing glutamate-mediated neuronal damage (109). IL-1β has been identified as mainly responsible for these alterations by increasing AMPA receptor activity. Inflammation-induced synaptic alterations in MS have also been investigated using a heterologous chimeric model. T-lymphocytes isolated from the peripheral blood of RR-MS patients exacerbated the glutamatergic transmission when incubated on mice brain slices (110). In particular, only lymphocytes from patients with acute inflammation, as evidenced by the presence of gadolinium-enhancing lesions at MRI, were able to induce synaptic alterations. Notably, co-incubation with etanercept, a TNF antagonist, prevented these alterations, confirming that TNF was mainly responsible for these findings (110).

### Inflammation and Corticospinal Excitability in MS

The role of inflammation on synaptic dysfunction in MS has been specifically addressed by some TMS studies. In relapsing MS patients, it has been shown to both reduce CSP duration and impair SICI compared to remitting patients (111). These results demonstrate that the relapsing phases could be characterized by cortical hyperexcitability, suggesting reduced GABAergic transmission similarly to as evidenced in animal models (106, 107). To explore the role of CSF inflammation on cortical excitability, different TMS measures have been correlated with the levels of specific proinflammatory molecules. In relapsing MS patients, elevated IL-1β signaling has been associated with increased ICF without effect on SICI (109). This finding has confirmed the main role of this molecule in altering synaptic functioning also in human MS by enhancing glutamatergic transmission (109). The involvement of this molecule in the excitotoxic degeneration has also been suggested by clinical studies, showing that CSF IL-1β detectability during remissions predicted greater prospective disability and neurodegeneration (112). Other inflammatory mediators have also been associated to altered synaptic transmission in relapsing MS patients. Regulated upon activation, normal T-cell expressed and secreted (RANTES) is a proinflammatory molecule regulating the leukocyte chemotaxis (113). Increased RANTES concentrations have been found in the CSF of MS patients with acute inflammation and correlated with both reduced SICI and increased ICF (114). Finally, incubating this molecule on mice hippocampal slices promoted hyperexcitability and excitotoxicity (114), confirming the role of RANTES as a central regulator of glutamatergic transmission (113).

Overall, these results indicate that exacerbated CSF inflammation negatively influences the disease course of MS, promoting synaptic hyperexcitability and neuronal damage. It has been proposed that neurodegeneration in progressive MS phenotypes could also result from inflammation-driven synaptic alterations. In fact, reduced SICI and enhanced ICF have been reported in SP-MS patients and have been related to enhanced disability (55). These findings suggest that glutamatergic excitotoxic damage could characterize the progressive MS phenotypes as demonstrated by *in vitro* studies showing hyperexcitability and enhanced neuronal damage induced by CSF collected from progressive MS patients, mediated by TNF (115). Conversely, anti-inflammatory cytokines, including IL-10 and IL-13, and neurotrophic factors may exert protective effects, reducing neurodegeneration and promoting a better disease course in EAE and MS (116–119). TMS studies have confirmed that anti-inflammatory molecules could reduce the synaptic alterations in MS. Accordingly, in RR-MS patients, the CSF levels of the anti-inflammatory molecule IL-13 have been associated with increased SICI, possibly contributing to restored inhibitory synaptic activity and limiting the impact of excitotoxicity. Notably, IL-13 CSF levels were also associated with reduced measures of neuronal and axonal damage and with increased amyloid-beta CSF concentrations, suggesting a protective role of this cytokine in MS (120).

## Conclusions and Future Perspectives

Various TMS protocols have been used to characterize the neurophysiological correlates of specific pathophysiological mechanisms, such as demyelination and neuronal loss, in different disease phases and phenotypes. These studies have contributed to better defining the neurophysiological basis of specific MS symptoms, particularly those not completely explained by conventional structural damage measures, such as fatigue and cognitive deficits. Alterations of corticospinal excitability and corticospinal tract conduction have been clearly linked to both demyelinating blocks and axonal damage. MEP latency and amplitude are the most frequently altered TMS measures in MS and have been consistently associated with disability, representing useful tools in clinical settings. Although TMS studies investigating intracortical excitability and effective connectivity have shown some association with specific pathophysiological mechanisms or disease phenotypes, some discrepancies suggest that alternative mechanisms should be involved.

Evidence from experimental studies suggests that inflammatory synaptopathy could represent an independent cause of synaptic dysfunction with important implications on disease course and prognosis. Inflammation, altering corticospinal excitability and connectivity in MS patients, could contribute to better explain the variability of TMS findings. Experimental models have clearly shown that inflammation exacerbates synaptic hyperexcitability, and TMS studies have confirmed an imbalance between excitatory and inhibitory transmission in MS patients. Hence, inflammation-driven synaptic hyperexcitability could be present since the early phases, could specifically characterize MS relapses, and could progressively increase during the disease course.

Having in mind that TMS measures represent the resulting effect of different anatomic and physiological factors, it is difficult to identify the contribution of specific mechanisms to TMS alterations seen in MS patients. Therefore, when cortical excitability measures are used to investigate MS pathophysiology, the role of specific confounding factors, including disease activity and phenotypes, ongoing therapies, and symptoms, such as fatigue, should be carefully considered. Further studies conducted in specific populations, such as patients with clinically isolated syndrome or with progressive MS, or combining TMS with structural and/or functional imaging data, could help to shed light on the specific role of demyelination, atrophy, and inflammation.

## Author Contributions

MS, EI, and DC contributed conception and design of the study, MS and EI wrote the first draft of the manuscript, FB, LG, ND, and DF wrote sections of the manuscript. All authors contributed to manuscript revision, read and approved the submitted version.

## Conflict of Interest

The authors declare the following potential conflicts of interest with respect to the research, authorship, and/or publication of this article: FB acted as Advisory Board members of Teva and Roche and received honoraria for speaking or consultation fees from Merck Serono, Teva, Biogen Idec, Sanofi, and Novartis and non-financial support from Merck Serono, Teva, Biogen Idec, and Sanofi. DC is an Advisory Board member of Almirall, Bayer Schering, Biogen, GW Pharmaceuticals, Merck Serono, Novartis, Roche, Sanofi-Genzyme, and Teva and received honoraria for speaking or consultation fees from Almirall, Bayer Schering, Biogen, GW Pharmaceuticals, Merck Serono, Novartis, Roche, Sanofi-Genzyme, and Teva. He is also the principal investigator in clinical trials for Bayer Schering, Biogen, Merck Serono, Mitsubishi, Novartis, Roche, Sanofi-Genzyme, and Teva. His preclinical and clinical research was supported by grants from Bayer Schering, Biogen Idec, Celgene, Merck Serono, Novartis, Roche, Sanofi-Genzyme and Teva. The funders had no role in the design of the study; in the collection, analyses, or interpretation of data; in the writing of the manuscript, or in the decision to publish the results. The remaining authors declare that the research was conducted in the absence of any commercial or financial relationships that could be construed as a potential conflict of interest.
